# Anosognosia for motor deficits in patients with left hemisphere lesions: a systematic review

**DOI:** 10.3389/fneur.2025.1681303

**Published:** 2025-10-29

**Authors:** Maura Simioni, Stefania Basilico, Martina Gandola

**Affiliations:** ^1^Department of Brain and Behavioral Sciences, University of Pavia, Pavia, Italy; ^2^NeuroMI—Milan Center for Neuroscience, University of Milano Bicocca, Milan, Italy; ^3^Cognitive Neuropsychology Center, ASST Grande Ospedale Metropolitano Niguarda, Milan, Italy

**Keywords:** anosognosia for motor deficit, anosognosia for hemiplegia, left hemispheric lesion, motor awareness, stroke

## Abstract

**Background:**

Patients with brain damage may deny the presence of their contralesional motor deficits. Some individuals may even claim they performed specific actions with the paralyzed limb, such as clapping hands. This well-known condition, called anosognosia for motor deficits, has been more frequently associated with right-brain lesions, primarily involving the posterior parietal cortex, the frontal cortex, and the insula. Instances of anosognosia for motor deficits in patients with left hemispheric lesions have also been described. However, less is known about the underlying mechanisms or differences in clinical manifestation.

**Methods:**

Following PRISMA guidelines, the present systematic review investigated the prevalence of anosognosia for motor deficits in patients with left-hemispheric brain lesions, focusing on its severity, clinical manifestation, and anatomical correlates. Moreover, we review adopted assessment methods and discuss the potential role of handedness and atypical hemispheric specialization in determining anosognosia for motor deficits. A comprehensive search across multiple databases up to the 28th of February 2025 identified 893 studies, with 25 included in the present study.

**Results:**

Reported prevalence of anosognosia for motor deficits in left brain-damaged patients ranged from 3.6 to 50% of assessed patients. These wide-ranging estimates may reflect the high heterogeneity in the tools adopted to assess both motor deficits and anosognosia, as well as in the diagnostic criteria employed to define anosognosia itself. Lesional data, when provided, showed a substantial overlap with the distributed network identified as the lesion substrate of anosognosia following right-hemisphere damage.

**Conclusion:**

Anosognosia for motor deficits following left-hemisphere lesions is less rare than previously assumed, thus challenging the hypothesis that the right hemisphere has an exclusive role in motor awareness. However, considering the sparsity and heterogeneity of current evidence, multicentric studies are required to better characterize the specific features of anosognosia associated with left-sided lesions and tackle unresolved issues such as the role of atypical hemispheric specialization.

## 1 Introduction

Anosognosia for motor deficits refers to a neuropsychological condition in which patients deny their motor impairment following brain injury. The term was coined by Babinski in 1914 (1) to describe the behavior of two patients with a right cerebral stroke and left hemiplegia who “were unaware of or seemed to be unaware” of the existence of the motor deficit that affected their limbs (1–4). In later years, the term anosognosia has been expanded to encompass awareness of a broader range of conditions, including hemianopia, language impairment, tactile perception, proprioception, and affective disturbances (5). Nevertheless, in the present review, we will focus on anosognosia for motor deficits, adhering to the original definition of the disturbance. Anosognosia ranges from anosodiaphoria [i.e., emotional indifference toward the deficits; (1)] to severe unawareness of contralateral motor deficits, in which patients do not acknowledge the motor impairment even when its presence is demonstrated through the neurological examination (for example, when asked to clap their hands). In these severe cases, patients may even claim to have performed actions that did not occur, showing false beliefs of movement (6, 7).

The question of whether anosognosia is lateralized in the brain was first raised by Babinski (1, 2), who, after observing the disorder in two patients with right-hemisphere lesions, posed the question: “Might anosognosia be specific to lesions of the right hemisphere?”. Since then, it has been widely accepted that anosognosia occurs more frequently after right hemisphere lesions (8–12), primarily involving the posterior parietal cortex, the frontal cortex, and the insula (7, 11, 13, 14), and most studies agree that its occurrence after left hemispheric lesions is considered relatively rare. Nevertheless, several cases of anosognosia following left hemisphere damage have been documented in the literature (see Table 1), challenging the notion that the phenomenon is exclusive to the right hemisphere.

**Table 1:** Assessment of motor impairment and anosognosia for motor deficits.

Study	Motor impairment			Anosognosia for motor deficits			
	Assessment	Severity	Affected limb	Assessment	*N* (%)	Severity	Affected limb
Sandifer, 1946 (55)	Clinical observation	Complete hemiplegia	Arm and leg	Clinical observation and informal interview	1 (100%)^a^	Severe	Arm
Nathanson et al., 1952 (10)	Clinical observation	Complete hemiplegia	n.r.	Anosognosia semi-structured interview (10)	9 (23%)^a^	n.r.	n.r.
Weinstein et al., 1964 (54)	Clinical observation	Complete hemiplegia	n.r.	Anosognosia semi-structured interview (54)	7 (100%)^a^	Severe (2 patients), moderate (3 patients), mild (2 patients)	n.r.
Green and Hamilton, 1976 (52)	Clinical observation	Complete hemiplegia	n.r.	Clinical observation	1 (4%)^a^	n.r.	Arm/leg
Cutting, 1978 (9)	Four level weakness scale (1 = slight; 2 = moderate; 3 = severe; 4 = total)	n.r. in AN patients; (3.8 in whole LBD sample)	Arm/leg	Anosognosia questionnaire (9)	3 (14%)^a^	n.r.	Arm/leg
Dronkers and Knight, 1989 (51)	Clinical observation	Paresis	Arm/leg	n.r.	1 (100%)	n.a.	n.a.
Cohen et al., 1991 (48)	Standard neurological examination (16)	Complete hemiplegia	Arm and Leg	Clinical observation and informal interview	1 (100%)^a^	Severe	Arm and leg
Starkstein et al., 1992 (12)	n.r.	Hemiparesis	n.r.	Anosognosia questionnaire [adapted from Bisiach et al. (16) and Cutting (9)]	3 (15.8%)	Moderate (score = 2/3)	n.r.
DeLuca, 1993 (50)	Clinical observation	n.r.	n.r.	n.r.	1 (100%)	n.a.	n.a.
Stone et al., 1993 (49)	Clinical observation	Complete hemiplegia	n.r.	Anosognosia questionnaire (9)	3 (5%)^a^	n.r.	n.r.
Grotta and Bratina, 1995 (53)	NIH stroke scale (56)	Arm and leg weakness	Arm and leg	Anosognosia structured questionnaire by Grotta and Bratina (53)	4 (50%)	n.a.	Arm and leg
Hartman-Maeir et al., 2001 (43)	Action research arm test (ARAT) (58)	Complete hemiplegia	Arm	Task choice method (60); awareness semi-structured interview (20)	4 (24%)^a^ TCM; None AI	n.r.	Arm
Hartman-Maeir et al., 2002 (44)	Standard neurological examination (16)	From mild hemiparesis to complete hemiplegia	Arm and leg	Awareness semi-structured interview (20)	3 (16%)^a^	Mild	Arm and leg
Beis et al., 2004 (39)	Scandinavian neurological stroke scale (57)	Complete hemiplegia	n.r.	Anosognosia structured interview (16)	5 (6%)^a^	n.r.	n.r.
Marcel et al., 2004 (22)	Medical research council motor scale (MRC-MS)	- MRC = 0–2:16 patients (73%); - MRC = 3–4: 6 patients (27%)	Leg	Awareness semi-structured interview (20); estimates of current ability on bilateral tasks (22)	2 (9%)^a^	n.r.	Leg
Baier and Karnath, 2005 (17)	Clinical ordinal scale (0 = no movement—5 = normal movement)	From mild hemiparesis to complete hemiplegia	Arm/leg	Anosognosia structured interview (16)	2 (3.57%)^a^	Moderate (score = 2/3)	Arm/Leg
Cocchini et al., 2009 (24)	Standard neurological examination (16)	Complete hemiplegia	Arm/leg	Anosognosia structured interview (21); VATAm (23)	12 (40%) VATAm; 2 (10%) AI^a^	Patient 1: moderate for UL (score = 2/3), mild for LL (score = 1/3); Patient 2: mild for both UL and LL (score = 1/3)	Arm/Leg
Robbins et al., 2009 (47)	Clinical observation	Complete hemiplegia	Leg	n.r.	1 (100%)^a^	n.r.	Leg
Moro et al., 2011 (46)	Clinical observation	Complete hemiplegia	Arm and leg	Anosognosia structured interview (21); estimates of current ability on bilateral tasks (22)	1 (100%)^a^	n.r.	Arm/leg
Cogliano et al., 2012 (41)	Not reported (clinical description)	Uncoordinated movements	Arm	Modified version of the estismates of current ability on bilateral tasks (22)	1 (100%)^a^	n.r.	Arm
Cocchini et al., 2013 (40)	Medical research council motor scale (MRC-MS)	n.r.	n.r.	VATAm (23)	5 (18%)^a^	n.r.	n.r.
Ronchi et al., 2013 (34)	Standard neurological examination (16)	Complete hemiplegia	Arm and leg	Anosognosia structured interview (16)	1 (100%)^a^	Severe (score = 3/3)	Arm/leg
Baier et al., 2014 (33)	Clinical ordinal scale (0 = no movement—5 = normal movement) (17)	Severe hemiparesis	Arm/leg	Anosognosia structured interview (16)	1 (2%)^a^	Severe (score = 3/3)	Arm
Formica et al., 2022 (42)	Standard neurological examination (16); Motricity index (MI) (59)	Hemiplegia (MI score = 32)	Arm/leg	VATAm (23)	1 (100%)^a^	Moderate (score = 18/36)	Arm/leg
Matsuyama et al., 2024 (45)	Medical research council motor scale (MRC-MS)	MRC = 1–2	Arm/leg	n.r.	1 (100%)	n.r.	Arm

n.r., not reported; AN, Anosognosia; LBD, Left Brain Damaged; NIH, National Institutes of Health; VATAm, Visual-Analogue Test for Anosognosia for Motor Impairment; TCM, Task Choice Method; UL, Upper Limb; LL, Lower Limb; AN, Anosognosia.

a Authors specify that patients present anosognosia for hemiplegia (AHP) and not general anosognosia for motor deficits.

In literature, the reported incidence of anosognosia for motor deficits in right-brain damaged patients varies considerably, ranging, for example, from 13% (15) to 58% (9). This variability may be attributed to several factors, such as differences in diagnostic criteria, patient selection, assessment methods, and the timing of evaluation (i.e., acute versus chronic phases). For example, when stringent diagnostic criteria are applied, such as not considering as anosognosic patients who fail to mention the paresis spontaneously but acknowledge it when directly questioned [i.e., score 1/3 in the Bisiach et al. scale (16)], the incidence drops considerably, ranging from 10% (17) to 32.43% (12). Concerning the incidence of anosognosia in Left Brain Damaged (LBD), the estimate is further complicated by the fact that many patients with extensive lesions compatible with the presence of anosognosia are excluded due to language problems and cannot be tested. In a previous influential review (18), the reported incidence of anosognosia in LBD patients ranges from 14% (9) to 30% (19), with a high variability between studies.

One of the most widely accepted explanations for this hemispheric asymmetry is that the prevalence of anosognosia following left-sided injury may be underestimated, due to the presence of co-occurring language impairments. These deficits may hinder patient evaluation, which typically relies on structured interviews [(16, 20–22), Appendix A] where patients are asked to provide verbal answers to general questions about the reason for their hospitalization or disorder-specific questions about their symptoms, or require a verbal estimation of the ability to perform unimanual or bimanual actions [(21, 22), Appendix B]. In this context, a significant breakthrough in the study of anosognosia in LBD patients was the introduction of the Visual Analogue Test (VATAm), which enables the assessment of anosognosia for motor deficits even in individuals with language impairments (23). This test has the advantage of being suitable for patients with language deficits, as it includes nonverbal stimuli (e.g., drawings of actions) and allows nonverbal responses using a Visual Analog Scale. Cocchini et al. (24) directly tested the hypothesis of a possible underestimation of anosognosia in LBD patients by comparing two different assessment methods to assess anosognosia: a traditional structured verbal interview (21) and the non-verbal VATAm test (23). Their results showed that using the structured interview led to the exclusion of a relatively high proportion of patients (i.e., 22/42 patients excluded: 52.4%), consistent with previous findings. In comparison, the exclusion rate dropped significantly (i.e., 12/42 patients excluded: 28.6%) when the VATAm test was used, allowing for the evaluation of 71% of the patients with LBD. Moreover, using the VATAm test, the prevalence of anosognosia for motor deficits increases significantly: the structured interview (21) identified anosognosia in only a minority of LBD patients (10%), whereas the VATAm test increased this to up to 40%. The use of non-verbal tools may enable the assessment of a larger number of patients and enhance the sensitivity of diagnosing anosognosia, particularly in patients with LBD.

Studies using the intra-carotid sodium amobarbital procedure (Wada Test) support the hypothesis of a non-exclusive role of the right hemisphere network in anosognosia for motor deficits (25–32). Although this procedure was traditionally used to assess hemispheric dominance for language and memory in patients with drug-resistant epilepsy, it also induces contralateral motor deficits, making it suitable for evaluating motor awareness. Following the injection of the barbiturate, the hemisphere ipsilateral to the infusion is temporarily inactivated, resulting in contralateral motor deficits. During the hemispheric inactivation, patients can be questioned about their motor abilities to determine whether they are aware of the resulting motor deficit. The majority of studies reported a right–left hemispheric asymmetry: the presence of anosognosia after inhibiting the right hemisphere ranges from 66% (29) to 100% (30, 31), while for the left hemisphere, it ranges from 0% (30) to 86% (28). In their seminal study, Gilmore and colleagues (30) reported a striking hemispheric asymmetry: all eight patients tested were able to recall their motor deficits when the left hemisphere was inhibited, whereas none of the patients were aware of the motor weakness when the right hemisphere was suppressed (right–left asymmetry: 100% vs. 0%). However, subsequent investigations painted a more complex picture, showing that unawareness was also frequently observed following left-hemisphere inhibition, with some studies reporting comparable [Dywan et al. (29), 66% for both right and left hemispheres inhibition] or slightly lower rates of anosognosia than those observed after right-hemisphere suppression [Durkin et al. (28), right–left asymmetry: 94% vs. 86%; Lu et al. (32), right–left asymmetry: 80% vs. 59%]. Overall, although all studies indicate a hemispheric asymmetry favoring the right hemisphere in the emergence of anosognosia during the Wada test, there is also consistent evidence that motor unawareness can arise following inactivation of the left hemisphere [see Table 7.2 in (5), page 128].

Other authors have suggested that the left hemisphere does not play a role in motor awareness, and cases of AHP in patients with left-sided lesions can occur due to reversed hemispheric lateralization.

This hypothesis is supported by the observation that many patients with left-sided lesions and anosognosia are left-handed, exhibit no or minimal language deficits after the lesion, and often present with right neglect, which supports the possibility of right hemispheric lateralization of language functions (i.e., reversed hemispheric lateralization). Baier et al. (33), for example, identified, one LBD patient (2%) out of a group of 66 patients who showed severe anosognosia for motor deficits. Despite the presence of severe right hemiparesis, the patient did not recognize her motor deficit, even when the neurological evaluation clearly demonstrated her inability to move, thus indicating the presence of a severe anosognosia [score 3/3 on the (16)]. The patient was right-handed and showed a large left hemispheric ischemic lesion involving the superior temporal gyrus, the angular gyrus, the insula, the inferior frontal gyrus, the postcentral gyrus, and the Rolandic operculum. She exhibited severe right hemiparesis (arm: 1/5; leg: 2/5), severe right-sided neglect, but not language deficits. Functional MRI examination revealed asymmetric brain activity in the right hemisphere during a sentence generation task. The authors concluded that the coexistence of -sided neglect, AHP, and right-sided lateralization of language functions suggests that the left hemisphere may not play an original role in motor awareness.

Similarly, Ronchi and coworkers (34) described an ambidextrous patient (G. B.) with severe anosognosia [score 3/3 on the Bisiach et al. (16) scale] for right hemiplegia with a large left cortical and subcortical lesion involving the temporoparietal junction. The patient also exhibited personal and extrapersonal neglect and somatoparaphrenia, generally associated with right hemisphere lesions. The application of Caloric Vestibular Stimulation (CVS) led to a remission of AHP (score pre-CVS: 3/3; score post-CVS: 0/3), which persisted for 2 days after stimulation and neglect. Despite the extensive lesion in the left hemisphere, the patient’s aphasic symptoms were mild, leading the authors to suggest that the patient’s language functions might be partially lateralized in the left hemisphere, with the possible involvement of the right hemisphere in language production and comprehension.

Although numerous reviews on anosognosia have been published, none to date have explicitly focused on motor unawareness following left-hemisphere lesions. The present review aims to fill this gap by providing a comprehensive overview of anosognosia for motor deficits in patients with left brain lesions, with particular attention to its prevalence, severity, clinical manifestation, assessment methods, and anatomical correlates.

## 2 Materials and methods

We followed the Preferred Reporting Items for Systematic Reviews and Meta-Analysis 2020 (PRISMA) guidelines (35) to guide the reporting and conducting of the present systematic review. Our research question was to determine the prevalence of anosognosia for motor deficits in patients with left-hemispheric brain lesions.

### 2.1 Search strategy and data sources

Two electronic databases (PubMed/Medline and PsycINFO) were searched on the same day (28 February 2025) to identify potentially relevant studies. The search strategy (Table 2) was first developed in PubMed and then adapted to be used in PsycINFO, including a specific combination of free text, exploded MeSH headings, and keywords to be found in title/abstract, combined with Boolean operators AND, OR and NOT, identifying two main components: (i) anosognosia, (ii) motor impairment. Screening of the reference list of included articles and consultation with experts in the field were also conducted to identify any additional relevant articles.

**Table 2:** A priori defined inclusion and exclusion criteria according to the SPIDER framework.

Search strategy	Details
Inclusion criteria	S: adult patients affected by left hemispheric brain lesions determining motor impairment (hemiplegia, hemiparesis) PI: anosognosia for motor deficits D: group studies and case reports reporting original data E: clinical assessment tools for anosognosia for motor deficits R: qualitative, quantitative or mixed method studies
Exclusion criteria	S: patients with right hemispheric or bilateral brain lesions PI: anosognosia for other neurological, cognitive and behavioral disorders; studies in which it is not clearly stated for which disorder patients display anosognosia; studies in which it is not possible to disentangle anosognosia for motor deficits vs. other neurological, cognitive and behavioral disorders D: studies reporting previously published data E: no restrictions R: studies not published as peer-reviewed, book, book chapter, thesis, protocol, no full-text papers (abstract, conference paper, letter, commentary, erratum, correction, editorial, note), reviews and meta-analyses
Language filter	English
Time filter	None
Database	PubMed/Medline, PsycINFO

SPIDER, Sample, Phenomenon of Interest, Design, Evaluation, Research type.

Inclusion and exclusion criteria were detailed based on Sample, Phenomenon of Interest, Design, Evaluation, and Research type (SPIDER) (36). The literature search was limited to studies published in English language, including human subjects, while no time filter was applied to the research. Studies including adult patients with left hemispheric brain lesions in which anosognosia for motor deficits was investigated were considered eligible.

### 2.2 Study selection, data extraction

The study selection process was carried out in two phases. The first screening was independently conducted by two authors (MS and MG) based on the title and abstract. Only eligible articles that passed this first phase were then evaluated in full text. At both stages, disagreements between reviewers were solved by discussion between the two authors; if controversy persisted, a third author was consulted (SB). Studies written by the same authors and referring to identical samples reporting the same number of participants were excluded.

Data extraction was then carried out independently by two authors (MS, MG) employing a data spreadsheet previously elaborated and agreed upon by the team, and pre-piloted on two randomly selected papers. Various qualitative and quantitative data were extracted, including: full reference details, study design, number of LBD patients enrolled, number of patients excluded for language disorders or other reasons, number of patients evaluated for the presence of anosognosia for motor deficits, sample characteristics, etiology, time since onset, tools employed to assess the presence and severity of motor deficits, tools used to evaluate the presence of anosognosia for motor deficits, prevalence and severity of both motor deficits and anosognosia for motor deficits, limb affected by motor deficit, prevalence of language deficits, prevalence of unilateral spatial neglect, and lesion location.

## 3 Results

### 3.1 Characteristics of included studies

**Figure 1:**
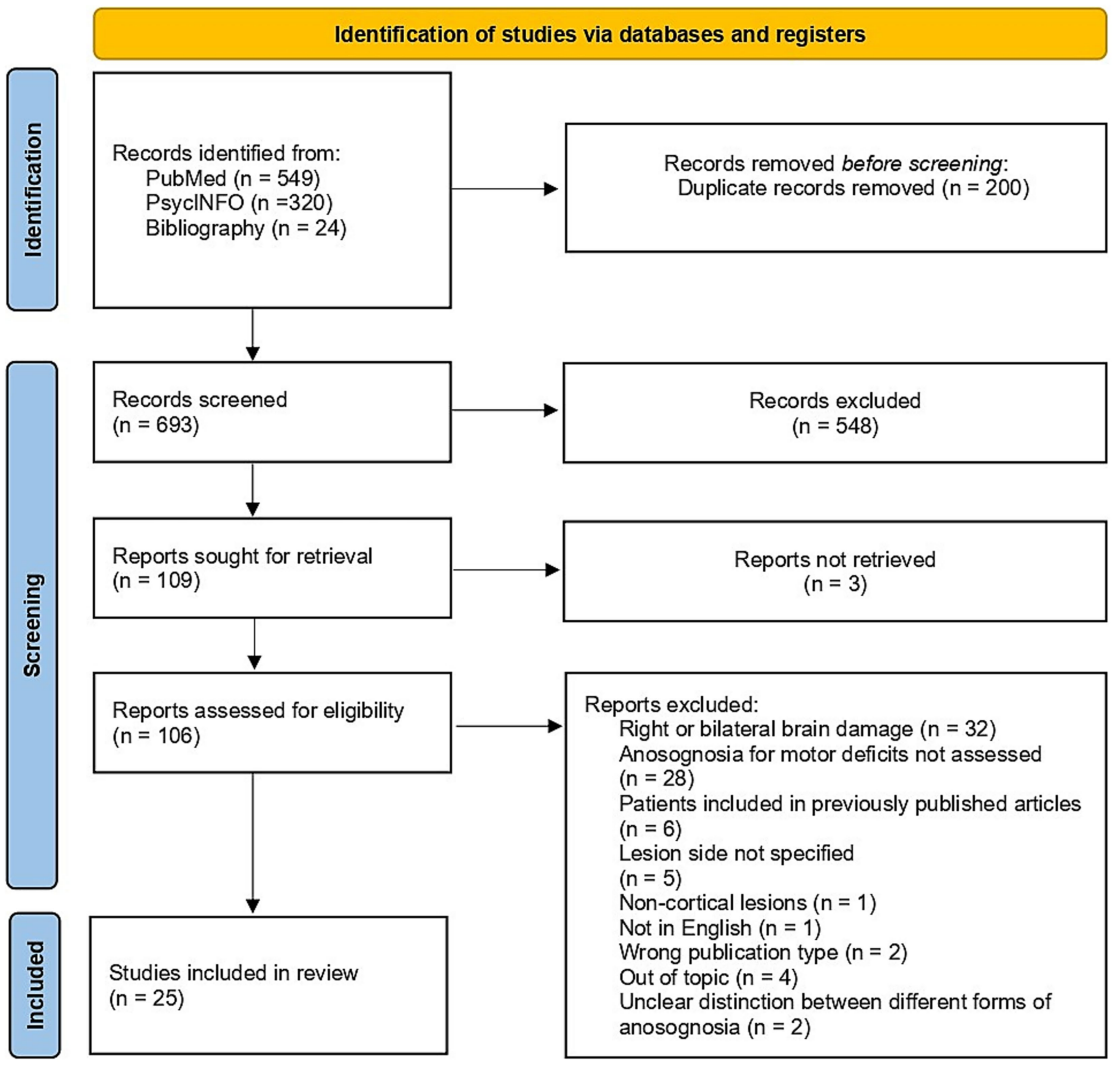
Flow diagram of the studies selection process [created employing Haddaway et al. (67) Shiny app for PRISMA 2020 compliant flow diagrams].

We identified 893 studies by searching the selected databases and listing references of relevant articles: 549 articles from PubMed, 320 from PsycINFO, and 24 from listing references of relevant articles. After removing duplicates, 693 records were retrieved. Papers were then screened: 584 records were excluded during the title and abstract screening, and three reports were not retrieved in full text. A total of 106 studies passed to the full text screening phase. Eighty-one were excluded for various reasons, resulting in 25 papers meeting our defined inclusion criteria and being included in the systematic review. Reasons for exclusion were the following: only Right Brain Damaged (RBD) patients, patients with bilateral lesions, articles not in English, anosognosia for motor deficits not assessed, wrong publication type, lesion side not reported, non-cortical lesions, out off-topic articles, patients included in previously published articles, inability to distinguish among different forms of anosognosia. More specifically, even though they were included in previous reviews on anosognosia for motor deficits, we excluded two studies by Weinstein and colleagues (37, 38) because they described patients with right-sided motor impairments but did not specify if the lesion was limited to the left hemisphere or was bilateral. Figure 1 shows the selection process.

Included studies (*n* = 25) were published between 1946 and 2024, with over 50% (*n* = 14, 56%) published after the year 2000 (17, 22, 24, 33, 34, 39–47). The large majority of studies (*n* = 13, 52%) were conducted in Europe (Italy = 5; Germany = 2; France = 3; UK = 3) (9, 17, 22, 24, 33, 34, 39–42, 46, 48, 49) and in the USA (*n* = 8, 32%) (10, 12, 47, 50–54). Moreover, two studies were conducted in Israel (43, 44) and one in Japan (45). Only in one case the country in which the study was conducted was not specified (55).

Among included studies (Table 3), 16 (64%) were group studies (9, 10, 17, 22, 24, 33, 39, 40, 43, 44, 46, 49, 52–55), 9 (36%) were case reports (34, 42, 45, 47, 48, 50, 51), and 2 (8%) were multiple-case series (12, 41). Overall, the sample sizes ranged from 1 to 102 patients, for a total sample size of 640 patients. Among them, 495 (77.34%) were actually tested for the presence of anosognosia for motor deficits. Indeed, 145 (22.65%) had to be excluded from the sample due to the presence of language deficits, which prevented the investigation of anosognosia for motor deficits, or due to other reasons [e.g., in Cocchini et al., (24)], 3 patients were excluded because they failed the Visual-Analogue Test for Anosognosia for Motor Impairment (VATAm) check questions.

**Table 3:** Sample characteristics.

Study	Study design	N LBD patients enrolled	Excluded from testing due to		Tested	Handedness	Pathology	Time since onset
			Language disorders	Other reasons				
Sandifer, 1946 (55)	Multiple single cases	1	n.a.	n.a.	n.a.	RH	Stroke	n.r.
Nathanson et al., 1952 (10)	Group study	63	24 (38%)	None	39 (62%)	n.r.	n.r.	n.r.
Weinstein et al., 1964 (54)	Group study	17^a^	None	None	17 (100%)	n.r.	Stroke, traumatic brain injury, tumor	n.r.
Green and Hamilton, 1976 (52)	Group study	25	None	None	25 (100%)	n.r.	n.r.	n.r.
Cutting, 1978 (9)	Group study	52	30 (58%)	None	22 (42%)	LH	Stroke/tumor	<8 days
Dronkers and Knight, 1989 (51)	Single case	1	n.a.	n.a.	n.a.	LH	Stroke	24 days
Cohen et al., 1991 (48)	Single case	1	n.a.	n.a.	n.a.	RH	Stroke	Range 1–21 days
Starkstein et al., 1992 (12)	Group study	19	n.r.	n.r.	19 (100%)	n.r.	Stroke	Range 1–12 days
DeLuca, 1993 (50)	Single case	1	n.a.	n.a.	n.a.	LH	Stroke	3 timepoints: 0 days, 66 days, 110 days
Stone et al., 1993 (49)	Group study	102	46 (45%)	n.a.	56 (55%)	n.r.	Stroke	n.r.
Grotta and Bratina, 1995 (53)	Group study	8	n.r.	n.r.	8 (100%)	n.r.	Stroke	n.r.
Hartman-Maeir et al., 2001 (43)	Group study	17	None	None	17 (100%)	RH	Stroke	M = 42 (SD = 13.55) days
Hartman-Maeir et al., 2002 (44)	Group study	24	n.r.	n.r.	24 (100%)	RH	Stroke	M = 43.33 (SD = 15.21) days
Beis et al., 2004 (39)	Group study	89	11 (12%)	None	78 (88%)	65 (83.2%) RH; 13 (16,8%) LH	Stroke	M = 10.8 (SD = 12.4) weeks
Marcel et al., 2004 (22)	Group study	22	n.r.	n.r.	22 (100%)	n.r.	Stroke	M = 79.1 (SD = 155) days
Baier and Karnath, 2005 (17)	Group study	56	n.r.	None	56 (100%)	n.r.	Stroke	Range 0–15 days
Cocchini et al., 2009 (24)	Group study	42	9 (21.45%) whole study; 4 (9.5%) SI	3 (1,5%) VATAm^b^	20 (%) SI; 30 (%) VATAm	n.r.	Stroke	M = 73.8 (SD = 46) days
Robbins et al., 2009 (47)	Single case	1	n.a.	n.a.	n.a.	RH	Stroke	n.r.
Moro et al., 2011 (46)	Group study	1	n.a.	n.a.	n.a.	LH corrected to RH	Stroke	177 days
Cogliano et al., 2012 (41)	Multiple single cases	1	n.a.	n.a.	n.a.	RH	Stroke	4 timepoints: 2, 7, 19 and 24 months
Cocchini et al., 2013 (40)	Group study	28	None	None	28 (100%)	n.r.	Stroke or Traumatic Brain Injury	41.2 (SD = 9.3, range 1–306) weeks
Ronchi et al., 2013 (34)	Single case	1	n.a.	n.a.	n.a.	Ambidextrous	Stroke	46 days
Baier et al., 2014 (33)	Group study	66	2 (33%)	None	44 (66.7%)	n.r.	Stroke	M = 5 (SD = 2) days
Formica et al., 2022 (42)	Single case	1	n.a.	n.a.	n.a.	RH	Stroke	10 days
Matsuyama et al., 2024 (45)	Single case	1	n.a.	n.a.	n.a.	LH corrected to RH	Stroke	3 days

N, Number; LBD, left brain damaged; n.r., not reported; n.a., not applicable; M, Mean; SD, Standard Deviation; SI, Structured Interview; VATAm, Visual-Analogue Test for Anosognosia for Motor Deficits; RH, Right-Handed; LH, Left-Handed; M, mean; SD, standard deviation.

a We only included patients for which lesion side was explicitly stated.

b Excluded from assessment due to failed check questions of the VATAm test.

### 3.2 Prevalence of anosognosia for motor deficits

Considering group studies only (*n* = 16), the reported prevalence of anosognosia for motor deficits in LBD patients ranged from 3.6 to 50% of assessed patients (see Table 1). Taking into account all included studies, among the patients effectively tested for the presence of anosognosia for motor deficits, the prevalence of the disorder ranges from 12.10 to 14.95%, depending on considered assessment method and diagnostic criteria. Indeed, two studies [i.e., (24, 43)] compared diagnostic accuracy of different assessment tools, leading to a varying degree of prevalence rate.

### 3.3 Assessment of motor deficits

Among the included studies motor deficits were evaluated with a wide variety of methods (see Table 1), ranging from simple clinical observation (10, 12, 41, 46, 47, 49–52, 54, 55), to the employment of more structured clinical ordinal scales, that classify patients based on the severity of their motor impairment. Several studies employed standardized scales, such as the Standard Neurological Assessment described by Bisiach et al. (16) (employed in 24, 34, 42, 43, 44, 48), the four-level weakness scale proposed by Cutting (9) and the clinical ordinal scales described by Baier and Karnath (17, 33). Other studies chose to employ standardized assessment tools like the NIH Stroke Scale (53, 56), the Scandinavian Neurological Stroke Scale (39, 57), the Action Research Arm Test (43, 58), the Motricity Index (42, 59) or the Medical Research Council Motor Scale (22, 40, 45).

### 3.4 Assessment and definition of anosognosia for motor deficits

Table 1 summarizes the assessment methods reported in the reviewed articles for evaluating anosognosia and motor deficits. The approaches used to investigate anosognosia for motor deficits varied widely, ranging from basic clinical observation (48, 52) to more structured and standardized approaches. Early attempts to assess anosognosia for motor deficits included semi-structured interviews that relied heavily on clinicians’ subjective judgment (10, 54). Subsequent studies (22, 43) employed more standardized tools, such as the Anderson and Tranel (20) semi-structured interview, structured questionnaires, such as the one employed by Cutting (9) and Stone and colleagues (49), or the one proposed by Grotta and Bratina (53), and structured interviews, like the one described by Bisiach et al. (16) (employed in 12, 17, 33, 34, 39) or the one by Berti et al. (21) (employed in 24, 46). All these instruments share the common feature of directly addressing patients’ awareness of their motor impairment, asking general or specific questions on motor abilities, thus depending on the patients’ language abilities. Alternative tools were developed, which investigate patients’ implicit awareness of their motor impairment by asking them to judge their ability to perform unimanual or bimanual tasks, such as the *Estimates of Current Ability on Bilateral Tasks*, proposed by Marcel et al. (22) (employed also in 41, 46), or to choose between carrying out unimanual vs. bimanual tasks like the *Task choice method* described by Ramachandran (60) (employed in 43), or to rate how well they would accomplish activities of daily living that require bimanual actions (20, 23). Furthermore, to overcome the high dependence on patients’ language abilities, which is particularly relevant when assessing LBD patients, some studies employed the *Visual Analogue Test for Anosognosia for Motor Impairment* [VATAm, (23)]. This tool minimizes the reliance on language skills, enabling a more accurate assessment of anosognosia for motor deficits even when verbal questioning is not feasible due to aphasia (24, 40, 42).

### 3.5 Handedness and atypical brain specialization

An open question that has been investigated in LBD patients entails the potential relationship between anosognosia for motor deficits and handedness, given the tight link that has been classically reported between hand dominance and cognitive functions’ lateralization, especially language and motor awareness (61). Among the reviewed studies, 10 (40%) did not report any information about patients’ handedness (10, 17, 22, 24, 33, 40, 49, 52–54). In the remaining studies, 111 right-handed patients were reported (39, 41–44, 47, 48), 37 left-handed (9, 39, 50, 51), one ambidextrous (34), and two left-handed patients who had been corrected to be right-handed (45, 46).

### 3.6 Lesional data

In the studies reviewed, when anatomical information is provided, it indicates substantial overlap with the distributed network identified as the lesion substrate of anosognosia following right-hemisphere damage, involving both cortical and subcortical regions located in the frontal (24, 33, 42, 43, 51, 53), parietal (24, 33, 43, 53) and temporal areas (33, 42, 53) or in the fronto-temporo-parietal (46) or the temporo-parietal junction (34). Regions frequently involved were also the insula (33, 42), the basal ganglia (24, 46, 50, 53), the internal capsule (24, 43, 50, 51, 53), the corona radiata (45), the thalamus (24, 50, 52), the centrum semiovale (50, 51) and the caudate nucleus (51) (See Table 4). The lesion data are reported for descriptive purposes only, as a quantitative analysis of the available lesion data was not possible due to the limited anatomical data included in the reivewed papers.

**Table 4:** Lesion location in patients with anosognosia for motor deficits.

Study	Cortical						Subcortical							
	F	T	P	O	Ins.	MCA	Thal.	B. G.	I. C.	C. R.	W. M.	C. S.	Unc.	CA
Weinstein et al., 1964 (54)		X	X	X										
Green and Hamilton, 1976 (52)							X							
Dronkers and Knight, 1989 (51)	X							X	X			X		
Cohen et al., 1991 (48)						X								X
DeLuca, 1993 (50)							X	X	X			X	X	
Grotta and Bratina, 1995 (53)	X	X	X					X	X					
Hartman-Maeir et al., 2001 (43)	X		X						X		X			
Cocchini et al., 2009 (24)	X		X				X	X	X					
Robbins et al., 2009 (47)	X													
Moro et al., 2011 (46)	X	X	X					X						
Cogliano et al., 2012 (41)			X	X										
Ronchi et al., 2013 (34)		X	X											
Baier et al., 2014 (33)	X	X	X		X									
Formica et al., 2022 (42)	X	X			X									
Matsuyama et al., 2024 (45)										X				

F, Frontal; T, Temporal; P, Parietal; O, Occipital; Ins., Insula; MCA, Middle Cerebral Artery territory; Thal., Thalamus; B. G., Basal Ganglia; I. C., Internal Capsule; C. R., Corona Radiata; W. M., White Matter; C. S., Centrum Semiovale: Unc., Uncus; CA, Choroidal Artery territory; the following studies did not report lesional data: Sandifer (55), Nathanson et al. (10), Cutting (9), Starkstein et al. (12), Stone et al. (49), Hartman-Maeir et al. (44), Beis et al. (39), Marcel et al. (22), Baier and Karnath (17), Cocchini et al. (40).

## 4 Discussion

The present systematic review summarized the available evidence on anosognosia for motor deficits in LBD patients. A total of 25 studies published between 1946 and 2024 were included, providing a comprehensive overview of the prevalence of this phenomenon and outlining the implications of employing different assessment methodologies. Moreover, the review explores the relationship between anosognosia for motor deficits, handedness, and the lateralization of cognitive functions.

Overall, considering only the 16 group studies included in the present review, the prevalence of anosognosia for motor deficits in LBD patients ranged from 3.6 to 50% of assessed patients. This wide range of incidence is consistent with findings in RBD patients, where reported prevalence rates varied between 33 and 58% (17, 18). The considerable variability observed across studies on anosognosia for motor deficits may reflect differences in the specific tools employed to assess both motor impairment and anosognosia, as well as in the diagnostic criteria used to define anosognosia for motor deficits.

Concerning the evaluation of motor deficits, there are two crucial aspects to consider. The first pertains to the variety of assessment methods used in the literature, while the second concerns the criteria adopted by authors for including patients in the evaluation of motor awareness. Regarding the first aspect, there is considerable heterogeneity in how motor deficits are assessed across studies. The tools used vary not only in precision and susceptibility to risk of bias, but more importantly, they differ in their focus (i.e., the specific aspect of motor impairment that is being assessed) and in how they define motor impairment itself. Indeed, some studies evaluated patients based on mere clinical observation (10, 12, 41, 46, 47, 49–52, 54, 55), making the classification of patients highly susceptible to observer-related bias. Other studies, instead, used various structured clinical ordinal scales (9, 16, 17), which classify patients in discrete categories based on the severity of motor impairment, or standardized assessment tools such as the NIH Stroke Scale (56), the Scandinavian Neurological Stroke Scale (57), the Action Research Armtest (58), or the Medical Research Council Motor Scale. These tools assess different aspects of motor impairment ranging from the ability to perform gross (9, 16, 17, 57, 58) or fine movements (58), to analysis of muscle strength [(56, 58); Medical Research Council Motor Scale]. Due to the employment of such heterogeneous instruments, the reported prevalence of motor impairments varies according to the strictness and focus of the specific assessment method used. Concerning the second aspect, i.e., the criteria used for including patients in the evaluation of AHP, some authors included only patients with complete hemiplegia (10, 39) to avoid ambiguous interpretations of responses to questions related to the motor impairment [see also Berti et al. (21)]. The adoption of such a strict criterion aims to ensure that the denial of the deficit cannot be attributed to a legitimate uncertainty about the severity of the motor impairment. In contrast, other authors have assessed unawareness in patients with hemiparesis, mild weakness, or incomplete motor deficits (19, 41). In these latter cases, the object of unawareness is not the impossibility of moving a limb (as in the case of a complete hemiplegia) but rather a more generic deficit, which may be harder for the patient to quantify. A limitation of the current reviewed literature is that the severity of the motor deficit for which awareness is being assessed is often not specified.

A similar argument applies to the choice of the assessment tool employed for evaluating anosognosia, which can significantly affect the reported prevalence. Indeed, assessment instruments differ not only in their structure but also in their complexity, sensitivity, focus, and reliance on patients’ residual language abilities. The first tools proposed to assess anosognosia for motor deficits [e.g., (10, 54)] were less structured and relied heavily on the clinician’s subjective judgement, while subsequent semi-structured (20) or structured questionnaires (9, 53) and interviews (16, 21) were developed to supply clinicians and researchers with more standardized methods that guide the diagnosis more systematically.

Another key difference among these assessment methods regards the focus of each tool. Indeed, while some directly assess the patients’ awareness of their motor impairment (9, 10, 16, 21, 53, 54), others target patients’ awareness of their ability to perform unimanual or bimanual actions (e.g., comb hair, tie a knot, clap hands, jump) (22, 60) or activities of daily living that require bimanual actions [see for example the post-test questions in Anderson and Tranel (20), page 339; Della Sala et al. (23)]. Furthermore, the above-mentioned instruments are mainly questionnaires or interviews, which rely deeply on patients’ explicit judgment; other tools, such as the *Task Choice Method* proposed by Ramachandran (60), represent implicit forms of assessing anosognosia for motor deficits, based on patients’ actual motor behavior. These differences have direct consequences on the reported prevalence rates, as demonstrated by studies comparing multiple assessment tools [e.g., (24, 43)].

Another critical aspect to consider, especially when considering LBD patients, concerns the degree of dependency of some tools on patients’ residual language abilities. Indeed, as highlighted by Della Sala et al. (23), the vast majority of instruments available for assessing anosognosia for motor deficits largely rely on language skills, thereby hindering the possibility of adequately evaluating patients with aphasia or even those with confined language impairments. The presence of language deficits is indeed one of the main exclusion criteria from studies focusing on the assessment of anosognosia for motor deficits, leading to a probable underestimation of the frequency of anosognosia for motor deficits in LBD patients. For this reason, instruments like the VATAm (23) were developed to minimize reliance on language skills and thus enable accurate assessment of anosognosia for motor deficits even when verbal questioning is impractical.

Finally, as already pointed out by previous studies (17), one of the key issues influencing the reported frequency of anosognosia for motor deficits across the literature, not only in LBD but also RBD patients, regards the definition of anosognosia for motor deficits (17, 18). Indeed, considering a patient as affected by anosognosia for motor deficits if they fail to report the presence of a motor deficit spontaneously, but then acknowledge such impairment after being questioned explicitly about it [i.e., score = 1 on the Bisiach et al. (16) structured interview], significantly increases the number of patients that can be diagnosed with anosognosia for motor deficits. However, as discussed by Baier and Karnath (17), those patients are not entirely unaware of their deficit: they fail to report it spontaneously, perhaps considering it less prominent if compared to other cognitive and neurological disorders. Therefore, the authors propose considering as truly affected by anosognosia for motor deficits only those patients who, when specifically asked about the strength of their limbs, insist on denying the presence of motor impairment. Applying this stricter definition of anosognosia for motor deficits leads to a more homogeneous incidence of the disorder across the published articles (17, 18).

Concerning the relationship between anosognosia for motor deficits, handedness and atypical brain organization, some authors (33, 34, 45, 50) suggested that individuals exhibiting anosognosia for motor deficits after LBD often present reversed language lateralization, which is more commonly observed in left-handed than in right-handed individuals (61). Matsuyama et al. (45) hypothesized atypical language lateralization in their patient (originally left-handed and corrected to employ the right hand during her childhood) because, despite a stroke affecting regions in the left hemisphere that are usually involved in language processes, she did not exhibit any language disturbance. Similarly, DeLuca (50) described a left-handed patient with anosognosia for motor deficits and extrapersonal neglect who, despite a large lesion in the left hemisphere, showed expressive and receptive language functions within normal limits. Baier et al. (33), by contrast, used fMRI language tasks in a right-handed patient who, despite a large hemispheric lesion, did not display any aphasic symptoms. The fMRI investigation clearly showed widespread activations in the right hemisphere during language tasks, suggesting that the network responsible for both speech production and comprehension was lateralized to the right hemisphere. On a slightly different note, Ronchi and colleagues (34) reported the case of an ambidextrous patient with anosognosia for motor deficits, extrapersonal neglect, and mild aphasic symptoms, suggesting a potential atypical lateralization of cognitive functions as well.

Despite these observations, the link between language lateralization, handedness and anosognosia for motor deficits is far from being clearly outlined. Indeed, Pedersen et al. (62) specifically tested this association by investigating whether there was a relationship between the presence of anosognosia for motor deficits and handedness in LBD patients, without finding any significant association. Indeed, in their sample, 95% of patients with anosognosia for motor deficits were right-handed, as were 95% of those without anosognosia. Similarly, among all the patients included in the present review for whom data on handedness was available, 110 (73.33%) out of 150 were right-handed. These findings highlight the need for further investigations to clarify this unresolved issue.

Finally, regarding the interhemispheric lesion site associated with anosognosia following left-brain damage, no large-scale studies to date have specifically investigated this topic or compared the lesion substrate of left-brain-damaged patients with and without anosognosia, as has been done in cases of right-hemisphere lesions. Across the reviewed studies that comprised anatomical data, a considerable overlap emerges with the lesion substrates associated with anosognosia following right-hemisphere damage, involving both cortical and subcortical structures within the frontal (24, 33, 42, 43, 51, 53), parietal (24, 33, 43, 53) and temporal lobes (33, 42, 53) as well as in the fronto-temporo-parietal (46) or the temporo-parietal junction (34). Frequently affected regions were also the insula (33, 42), the basal ganglia (24, 46, 50, 53), the internal capsule (24, 43, 50, 51, 53), the corona radiata (45), the thalamus (24, 50, 52), the centrum semiovale (50, 51) and the caudate nucleus (51). Nevertheless, the reported findings are highly heterogeneous, and the lack of systematic reports and group analyses precludes more definitive conclusions regarding the interhemispheric localization of anosognosia associated with lesions in the left hemisphere.

Overall, considering the relatively small sample size of the included studies, the high heterogeneity of assessment methods and diagnostic criteria for both motor deficits and anosognosia, and also the variety of inclusion criteria, which sometimes led to the exclusion of patients with aphasia from testing, it is evident that further investigation on anosognosia for motor deficits in LBD patients is needed, with the aim of addressing some unresolved issues with a more systematic approach. First, as suggested by the present review, anosognosia for motor deficits in LBD patients occurs more frequently than previously assumed. Therefore, its potential occurrence should be carefully assessed during the clinical evaluation of LBD patients. Indeed, the under-recognition of this condition in LBD patients may negatively affect their recovery, as it is associated with poorer outcomes due to decreased treatment compliance, motivation and engagement in rehabilitation programs (63–66). For these reasons, an accurate and timely diagnosis is crucial to deliver tailored interventions to affected patients.

Therefore, comprehensive studies on both RBD and LBD patients are necessary to obtain a more accurate estimate of the prevalence of this phenomenon. For this purpose, new systematic investigations are needed that employ standardized and detailed assessment tools for evaluating motor deficits, capable of differentiating between complete hemiplegia, hemiparesis, and varying degrees of motor impairment or weakness. Furthermore, when assessing anosognosia for motor deficits, adopting an evaluation method that allows the testing of anosognosia also in patients with language impairment would be recommended. Additionally, a more homogeneous definition of the phenomenon itself is needed, as, heterogeneous diagnostic criteria significantly influence the reported prevalence of anosognosia for motor deficits. Finally, to examine the hypothesis of an association between anosognosia for motor deficits, handedness, and atypical language lateralization, a systematic investigation of the prevalence of language impairments in LBD patients both with and without anosognosia together with the adoption of fMRI language tasks to map brain areas involved in language processes is needed to provide a clearer picture of the possible link between these phenomena.

## 5 Conclusion

In conclusion, our review suggests that anosognosia following left-hemisphere lesions is less rare than previously assumed. These findings challenge the hypothesis that the right hemisphere has an exclusive role in motor awareness. At the same time, considering the current evidence, multicentric studies are required to collect data from larger samples and better characterize the specific features of anosognosia associated with left-sided lesions. Such studies should also explore the relationship between anosognosia for right-hemiplegia and other neurological deficits (e.g., tactile imperception, proprioceptive deficits, etc.) and investigate the underlying neural substrates, which remain largely unexplored.

## Data Availability

The original contributions presented in the study are included in the article/supplementary material, further inquiries can be directed to the corresponding author.
